# Meta-analysis of epigenome-wide association studies of cognitive abilities

**DOI:** 10.1038/s41380-017-0008-y

**Published:** 2018-01-08

**Authors:** Riccardo E. Marioni, Allan F. McRae, Jan Bressler, Elena Colicino, Eilis Hannon, Shuo Li, Diddier Prada, Jennifer A Smith, Letizia Trevisi, Pei-Chien Tsai, Dina Vojinovic, Jeannette Simino, Daniel Levy, Chunyu Liu, Michael Mendelson, Claudia L. Satizabal, Qiong Yang, Min A. Jhun, Sharon L. R. Kardia, Wei Zhao, Stefania Bandinelli, Luigi Ferrucci, Dena G. Hernandez, Andrew B. Singleton, Sarah E. Harris, John M. Starr, Douglas P. Kiel, Robert R. McLean, Allan C. Just, Joel Schwartz, Avron Spiro, Pantel Vokonas, Najaf Amin, M. Arfan Ikram, Andre G. Uitterlinden, Joyce B. J. van Meurs, Tim D. Spector, Claire Steves, Andrea A. Baccarelli, Jordana T. Bell, Cornelia M. van Duijn, Myriam Fornage, Yi-Hsiang Hsu, Jonathan Mill, Thomas H. Mosley, Sudha Seshadri, Ian J. Deary

**Affiliations:** 1 Centre for Cognitive Ageing and Cognitive Epidemiology, University of Edinburgh, Edinburgh, UK; 2 Centre for Genomic and Experimental Medicine, Institute of Genetics and Molecular Medicine, University of Edinburgh, Edinburgh, UK; 3 Institute for Molecular Bioscience, University of Queensland, Brisbane, QLD Australia; 4 Queensland Brain Institute, University of Queensland, Brisbane, QLD Australia; 5 Human Genetics Center, School of Public Health, University of Texas Health Science Center at Houston, Houston, TX USA; 6 Columbia University Mailman School of Public Health, New York, NY USA; 7 Icahn School of Medicine at Mount Sinai, New York, NY USA; 8 University of Exeter Medical School, Exeter, UK; 9 Department of Biostatistics, Boston University School of Public Health, Boston, MA USA; 10 Instituto Nacional de Cancerologia, Mexico City, Mexico; 11 Department of Epidemiology, School of Public Health, University of Michigan, Ann Arbor, MI USA; 12 Survey Research Center, Institute for Social Research, University of Michigan, Ann Arbor, MI USA; 13 Harvard Medical School, Boston, MA USA; 14 Department of Twin Research and Genetic Epidemiology, King’s College London, London, UK; 15 Department of Biomedical Sciences, Chang Gung University, Taoyuan City, Taiwan; 16 Division of Allergy, Asthma, and Rheumatology, Department of Pediatrics, Chang Gung Memorial Hospital, Linkou, Taiwan; 17 Department of Epidemiology, Erasmus University Medical Center, Rotterdam, The Netherlands; 18 Department of Data Science, School of Population Health, University of Mississippi Medical Center, Jackson, MS USA; 19 MIND Center, University of Mississippi Medical Center, Jackson, MS USA; 20 Framingham Heart Study, Framingham, MA USA; 21 Population Sciences Branch, National Heart, Lung, and Blood Institute, National Institutes of Health, Bethesda, MD USA; 22 Boston University School of Medicine, Boston, MA USA; 23 Department of Cardiology, Boston Children’s Hospital, Boston, MA USA; 24 Department of Neurology, Boston University School of Medicine, Boston, MA USA; 25 Division of Public Health Sciences, Fred Hutchinson Cancer Research Center, Seattle, WA USA; 26 Geriatric Unit, Azienda Sanitaria di Firenze, Florence, Italy; 27 Clinical Research Branch, National Institute on Aging, Baltimore, MD USA; 28 Laboratory of Neurogenetics, National Institute on Aging, National Institutes of Health, Bethesda, MD USA; 29 Alzheimer Scotland Dementia Research Centre, University of Edinburgh, Edinburgh, UK; 30 Hebrew SeniorLife Institute for Aging Research, Boston, MA USA; 31 Harvard T.H. Chan School of Public Health, Boston, MA USA; 32 Boston University Schools of Public Health and Medicine, Boston, MA USA; 33 VA Boston Healthcare System, Boston, MA USA; 34 Department of Neurology, Erasmus University Medical Center, Rotterdam, The Netherlands; 35 Departments of Radiology, Erasmus University Medical Center, Rotterdam, The Netherlands; 36 Department of Internal Medicine, Erasmus University Medical Center, Rotterdam, The Netherlands; 37 Brown Foundation Institute of Molecular Medicine, McGovern Medical School, University of Texas Health Science Center at Houston, Houston, TX USA; 38 Broad Institute of MIT and Harvard, Cambridge, MA USA; 39 Department of Medicine, Division of Geriatrics, University of Mississippi Medical Center, Jackson, MS USA; 40 Glenn Biggs Institute of Alzheimer and Neurodegenerative Diseases, University of Texas Health Sciences Center, San Antonio, TX USA; 41 Department of Psychology, University of Edinburgh, Edinburgh, UK

## Abstract

Cognitive functions are important correlates of health outcomes across the life-course. Individual differences in cognitive functions are partly heritable. Epigenetic modifications, such as DNA methylation, are susceptible to both genetic and environmental factors and may provide insights into individual differences in cognitive functions. Epigenome-wide meta-analyses for blood-based DNA methylation levels at ~420,000 CpG sites were performed for seven measures of cognitive functioning using data from 11 cohorts. CpGs that passed a Bonferroni correction, adjusting for the number of CpGs and cognitive tests, were assessed for: longitudinal change; being under genetic control (methylation QTLs); and associations with brain health (structural MRI), brain methylation and Alzheimer's disease pathology. Across the seven measures of cognitive functioning (meta-analysis n range: 2557–6809), there were epigenome-wide significant (*P* < 1.7 × 10^-8^) associations for global cognitive function (cg21450381, *P* = 1.6 × 10^-8^), and phonemic verbal fluency (cg12507869, *P* = 2.5 × 10^-9^). The CpGs are located in an intergenic region on chromosome 12 and the *INPP5A* gene on chromosome 10, respectively. Both probes have moderate correlations (~0.4) with brain methylation in Brodmann area 20 (ventral temporal cortex). Neither probe showed evidence of longitudinal change in late-life or associations with white matter brain MRI measures in one cohort with these data. A methylation QTL analysis suggested that rs113565688 was a *cis* methylation QTL for cg12507869 (*P* = 5 × 10^-5^ and 4 × 10^-13^ in two lookup cohorts). We demonstrate a link between blood-based DNA methylation and measures of phonemic verbal fluency and global cognitive ability. Further research is warranted to understand the mechanisms linking genomic regulatory changes with cognitive function to health and disease.

## Background

Cognitive function is an important predictor of health outcomes and mortality [1–4]. Whether this is due to differences in health literacy and lifestyle choices or if there is a biological predisposition is not clear [5]. The complex balance between genetic and environmental contributions to cognitive function is poorly understood [6]. Epigenetic modifications may provide insight into the link between cognitive function, perturbed biological pathways and relevance for lifelong health.

Molecular genetic studies of unrelated individuals show that around 30% of the variance in general cognitive function can be explained by common genetic polymorphisms (single-nucleotide polymorphisms: SNPs) and variants in linkage disequilibrium with them [7–9]. However, there are relatively few well-established individual SNP predictors of cognitive function and those that have been identified explain a very small proportion of the variance in cognitive test scores [8].

Epigenetic marks may help us better understand the interaction between genes, the environment, and health-related quantitative traits, such as cognitive function, and common disease outcomes [10, 11]. The epigenome helps to regulate genes via, for example, chemical modifications to DNA. DNA methylation typically refers to the addition of a methyl group to a cytosine nucleotide placed next to a guanine in the DNA sequence. The addition or removal of the methyl group is a dynamic process and can be tissue specific with, for example, different epigenetic signatures in blood and brain. The proportion of cytosines methylated at a specific CpG site can be partly explained by both genetics and lifestyle/environment or a combination of these [12]. Studies have examined the association between DNA methylation with genotype [13, 14], metabolic factors, such as body mass index [15, 16], and environmental factors, such as smoking [17]. However, no large-scale population-based studies have examined the association of cognitive function with DNA methylation in circulating leucocytes.

One aspect of note for epigenetic epidemiology studies of brain-related traits (cognitive functions, schizophrenia, depression, dementia, etc.) is tissue (and cellular) specificity. As brain samples are not likely to be available until post-mortem, a proxy tissue is an attractive possibility to be explored for building relevant epigenetic signatures. In epidemiological studies, the most likely candidate is blood, which, although its methylation patterns are often dissimilar to those in the brain [18, 19], they have still been linked to mental health traits [20–22]. Identifying robust methylomic differences in relation to cognitive traits may improve our ability to predict cognitive decline and better understand the mechanistic link between cognitive function and deleterious health outcomes.

Here, we examine, using a meta-analytic approach, the associations between blood-based DNA methylation and several individual tests of cognitive functions in up to 6809 healthy, older-aged adults. First we test which, if any, CpG probes are associated with individual cognitive functions at an epigenome-wide level. Then we investigate these probes to see if they are (1) under genetic control (methQTLs), (2) stable over time, (3) associated with structural brain-imaging measures, (4) associated with Alzheimer's disease case–control status or neuropathology, (5) associated with DNA methylation levels in different brain regions and (6) associated with blood-based gene expression.

## Methods

### Overview

Epigenome-wide association studies were performed in 11 independent cohorts for seven cognitive function phenotypes. The number of cohorts contributing to each of the seven tests of cognitive function ranged from 3 to 10 (Table S1). A sample-size-based meta-analysis of *Z*-scores was performed on the overlapping cohort summary output for each cognitive test.

### Cohorts

Nine of the eleven cohorts that contributed to the analysis included participants of European ancestry: Framingham Heart Study, InCHIANTI, Lothian Birth Cohort 1921, Lothian Birth Cohort 1936, MOBILIZE Boston, Normative Aging Study, Rotterdam Study (Rotterdam Bios and Rotterdam III) and Twins UK. The Atherosclerosis Risk in the Community (ARIC) and Genetic Epidemiology Network of Arteriopathy's (GENOA) cohorts included participants of African American ancestry. Details of each cohort are presented in Appendix 1.

### Cognitive measures

Scores from seven different cognitive tests were assessed:

1. Wechsler Logical Memory [23, 24] as a measure of verbal declarative memory. The sum of the immediate and delayed tasks was used.
2. Wechsler Digit Symbol Test [25] *or* Symbol Digit Modalities Test [26] *or* Letter Digit Substitution Test [27] as a measure of processing speed, hereafter referred to as Digit Test. The total number of correct answers in the allocated time period was used. The three tests listed above are highly correlated [28].
3. Semantic Verbal Fluency [29] as a measure of an aspect of executive function (animal naming - total score).
4. Phonemic Verbal Fluency [29] as a measure of an aspect of executive function (letter fluency - total score).
5. Trail Making Test Part B [30] as a measure of an aspect of executive function (Natural log (ln) of the time taken in seconds).
6. Boston Naming Test [31] *or* National Adult Reading Test [32] *or* any other measure of vocabulary. The total number of correct answers was assessed.
7. Mini-Mental State Examination (MMSE) [33] as a measure of general cognitive function. Individuals with a score of less than 24 out of 30 were excluded from the analysis.

With the exception of the MMSE scores, any cognitive score that fell above or below 3.5 standard deviations from the mean was set to the mean plus or minus 3.5 standard deviations, respectively. These analyses were performed within each cohort independently for each cognitive test. Full details of the tests available within each cohort are provided in Appendix 1.

### DNA methylation

Whole-blood DNA methylation was assessed in each cohort using the Illumina HumanMethylation450 BeadChips [34]. Quality control was performed according to cohort-specific thresholds, described in Appendix 1. The blood samples for DNA methylation and cognitive ability were measured concurrently.

### Structural brain imaging

1.5 T structural brain imaging was assessed in one of the participating epigenome-wide association study (EWAS) cohorts: The Lothian Birth Cohort 1936. Full details have been reported previously [35]. Here, we considered two measures of white matter connectivity—fractional anisotropy (directional coherence of water diffusion) and mean diffusivity (average magnitude of water diffusion)—that have been previously associated with cognitive function [36, 37].

### Gene expression

The association between DNA methylation and gene expression was assessed using the Affymetrix Human Exon 1.0 ST Array in one of the participating cohorts: The Framingham Heart Study. Methodological details are provided in Appendix 1.

### Ethics

Ethical permission for each cohort is described in Appendix 1. Written informed consent was obtained from all subjects.

### Statistical analysis

#### Epigenome-wide association testing

For each cognitive test, two linear regression models were considered—a basic-adjustment model and a full-adjustment model. Both models treated methylation at the CpG sites (untransformed methylation beta value) as the dependent variable with the cognitive test score as the independent predictor of interest. In the basic-adjustment model, covariates included age, sex, white-blood cell counts (either measured or imputed [38]), technical covariates such as plate, chip, array and hybridisation date, and, where required, genetic principal components to account for population stratification. In the fully adjusted model, the following additional covariate terms were included: a quadratic term for age, an age x sex interaction; smoking status (current, ever, never) and body mass index. The findings from the fully adjusted model were considered as the primary output. Measurement details for all variables are presented in Appendix 1. Age was standardised within cohort to mean 0, variance 1, to avoid potential model convergence issues. Individuals with prevalent dementia or clinical stroke (including self-reported) were excluded.

#### Quality control filtering

Prior to the meta analysis, all probes on sex chromosomes were removed along with non-CpG probes, and any cross-reactive probes as reported by Chen et al. [39]. Genomic correction was applied to any cohort-specific results file with an empirical lambda of more than 1. The total number of probes included in the meta-analysis for each cognitive trait ranged between 421,335 and 421,633.

#### Trait-specific meta-analysis

The primary analyses were conducted in R [40]; sample-size weighted meta-analyses were conducted in METAL [41]. Several significance thresholds were considered. The most liberal threshold was a within meta-analysis Benjamini-Hochberg false discovery rate of *Q* < 0.05. Next was a within meta-analysis Bonferroni corrected *P*-value threshold: 0.05/*n*_probes_max_ = 0.05/421,633 = 1.2 × 10^-7^. Finally, the most conservative threshold applied was a Bonferroni corrected *P*-value that also adjusted for the seven meta analyses: 0.05/(*n*_probes_*n_meta-analyses_) = 0.05/(421,633*7) = 1.7 × 10^-8^.

#### Summary meta-analysis combining all cognitive traits

Finally, a meta-analysis of the summary output from the seven meta analyses was conducted for the fully adjusted models using the CPASSOC software [42] in R. As the cohorts contributed to multiple EWAS, and as the as cognitive test scores are positively correlated [43], a correlation matrix of the CpG *Z*-scores for the seven cognitive traits was included to reduce the false-positive rate [42]. A test assuming heterogeneity was assumed and default input arguments were set.

#### Methylation quantitative trait loci

To determine if the significant EWAS findings (at the most conservative threshold of *P* < 1.7 × 10^-8^) were partly under genetic control, a methylation QTL analysis lookup was performed using data from the Lothian Birth Cohorts of 1921 and 1936 (combined *n* = 1366), and the Brisbane Systems Genetics Study (*n* = 614) [44]. The discovery and replication thresholds set in that study were *P* < 1 × 10^-11^ and *P* < 1 × 10^-6^, respectively, with the combined LBC cohorts acting as a discovery data set (*P* < 1 × 10^-11^) with BSGS as the replication study (*P* < 1 × 10^-6^) and vice versa. SNPs within 2 Mbp of a CpG site were labelled *cis* methylation QTLs, and only the most significant SNP for each CpG were considered.

#### Longitudinal change in methylation

For the significantly associated CpG probes identified in the meta-analyses, longitudinal data from the Lothian Birth Cohort 1936 were used to chart change in methylation at these CpGs between ages 70 and 76 years. Stability in methylation levels might be indicative of potential genetic control or a long-term fixed effect of differential cognitive function on the probe. Variability in methylation levels may be a by-product or cause of cognitive change over time. Methylation data were available on participants at ages 70 (*n* = 920), 73 (*n* = 800) and 76 (*n* = 618) years. Linear mixed models with random intercept terms, adjusting for sex, imputed white-blood cell counts and technical variables, were used to determine the rate of change over time (the coefficient for the fixed effect age variable in the model) for each probe.

#### Structural brain-imaging associations with methylation

As cognitive function is a brain-related phenotype, it was of interest to see if blood-based methylation signatures for cognitive function were related to brain-imaging measures. Structural MRI data and covariate information were also available in 552 participants at the second wave of the Lothian Birth Cohort 1936—data from only the first wave of the cohort were included in the EWAS. The top associations from the EWAS meta-analyses were assessed at the second wave of the Lothian Birth Cohort 1936 in relation to age- and sex-adjusted brain structural fractional anisotropy and mean diffusivity using linear regression models, adjusting for age, sex, imputed white cell counts and technical covariates.

#### Blood–brain methylation correlations

Lookup analyses of significant CpG sites were performed in published data sets for both blood and brain (prefrontal cortex, entorhinal cortex, superior temporal gyrus and cerebellum) based EWAS findings for Braak staging and Alzheimer's disease status [21]. A second lookup was performed using results from blood and Brodmann areas 7, 10 and 20 from post-mortem samples of 16 individuals [45].

#### Gene expression associations

Transcriptome-wide association studies (TWAS) were conducted in the Framingham Heart Study for any significant probes from the cognitive EWAS. Linear mixed effects models with expression of each gene as the dependent variable, methylation as exposure and identical covariates to the EWAS were considered. A Bonferroni correction was applied (*P* < 0.05/nprobes = 0.05/17,873 = 2.8 × 10^-6^) to identify statistically significant associations.

## Results

### Study sample characteristics

**Fig. 1:**
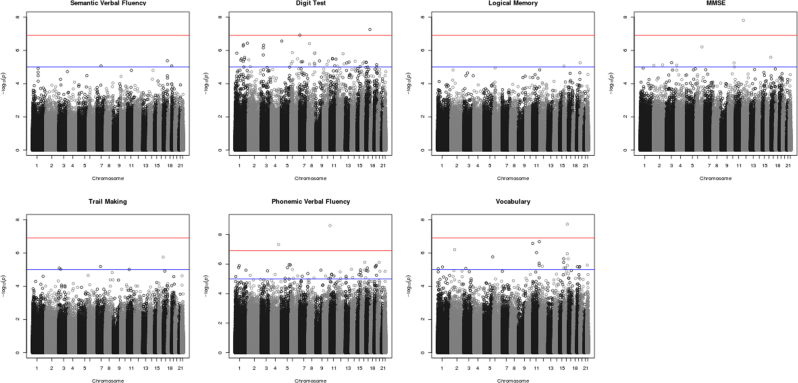
Meta-analysis EWAS Manhattan Plots for the seven cognitive tests—full adjustment models*. ^*^Models adjusted for age, sex, age x sex, age^2^, self-reported smoking status, body mass index, white-blood cell counts, technical covariates and principal components (population stratification)

Participants came from 11 cohorts—ranging in size from 219 to 2307 individuals (Q1–Q3: 435–920), with between 0 and 100% female participants (Q1–Q3: 52–65%), mean age ranged from 56 to 79 years (Q1–Q3: 60–73). Two of the cohorts (ARIC and GENOA) included participants of African American ancestry; all other cohorts included participants of European ancestry. The cohort-specific summary details for each cognitive test are presented in Supplementary Table 1. The basic-adjustment meta-analytic sample-size ranged from 2557 individuals for the Trail Making Test to 6809 individuals for the MMSE. Similar sample-sizes were observed for the fully adjusted models with the meta-analytic results presented in Fig. 1 and Table 1.

**Table 1:** Summary of meta-analysis results for the seven cognitive tests

	Cognitive test						
	Phonemic Verbal Fluency	MMSE^a^	Trail Making	Logical Memory	Vocabulary	Digit Test	Semantic Verbal Fluency
*N*_participants m1_	6405	6809	2557	2988	3013	4794	3678
*λ*_m1_	1.3	1.21	0.95	0.97	1.08	1.06	0.93
*N* loci_m1_	4	1	0	0	3	29	0
*N*_participants m2_	6390	6780	2549	2983	3007	4780	3658
*λ*_m2_	1.26	1.16	0.97	0.99	1.1	1.03	0.92
*N* loci_m2_	2	1	0	0	1	2	0

^a^*MMSE* Mini-Mental State Examination,

*m1* model 1, adjusted for age, sex, cell counts, technical covariates and population stratification (genetic principal components-cohort specific)

*m2* model 2, adjusted for model 1 covariates, smoking, body mass index, age^2^ and an age x sex interaction term

### Epigenome-wide association study model diagnostics

Heterogeneity was observed in the EWAS inflation statistics, both within and across cohorts (Supplementary Table 2). For example, the minimum and maximum lambda values in LBC1936 were 1.05 and 1.25, respectively. Prior to meta-analysis, within-cohort genomic correction was applied where lambda exceeded 1. The meta analysis genomic inflation statistics for the basic and fully adjusted models ranged from 0.93 to 1.30, and 0.92 to 1.26, respectively (Table 1).

### Epigenome-wide association study of seven cognitive traits

A list of the within-test epigenome-wide significant associations within a given cognitive test across both models are presented in Supplementary Table 3. Significant associations (*P* < 1.2 × 10^-7^) were observed in the basic and full adjustment models for Phonemic Verbal Fluency (*n* = 4 and *n* = 2), MMSE (*n* = 1 for both models), Vocabulary (*n* = 3 and *n* = 1), and Digit Test (*n* = 29 and *n* = 2). From the basic-adjustment model, significant CpGs were located in genes associated with, for example: alcohol metabolism (*ALDH2*, Digit Test, cg12142865) [46], smoking (*AHRR*, Digit Test, cg05575921) [17], inflammation (*CCR9* and *PRRC2A*, cg10475172 and cg14943908, respectively) [47, 48] and neurodegeneration through the beta-amyloid precursor protein interactor *GAPDH* (Digit Test, cg00252813) [49]. In the fully adjusted model, significant CpGs were located in genes associated with, for example: inflammation (*SOCS3*, Digit Test, cg18181703) [50], epithelial cell splicing (*ESRP2*, Vocabulary, cg04513006) [51] and transcription activation of NOTCH proteins (*MAML3*, Phonemic Verbal Fluency, cg16201957) [52]. No CpGs were significantly associated with the Trail Making, Logical Memory or Semantic Verbal Fluency tests. Methylation at cg21450381 was not associated with any of the six other cognitive traits in the fully adjusted meta-analytic results at a nominal significance threshold of *P* < 0.05 (Table 2). However, cg12507869 was associated with lower scores for both Logical Memory (*P* = 0.043) and Vocabulary (*P* = 9.4 × 10^-5^).

**Table 2:** Lookup of top EWAS associations across all cognitive tests in the fully adjusted models. The *P*-values for the initial EWAS associations at *P *< 1.7x10^-8^ are highlighted in bold

Cognitive Test	*N*	*Z*	*P*
Digit Test			
cg21450381	4780	0.51	0.61
cg12507869	4780	-1.44	0.15
Vocabulary			
cg21450381	3007	-0.61	0.54
cg12507869	3007	-3.91	9.4x10^-5^
Semantic Verbal Fluency			
cg21450381	3658	-1.11	0.27
cg12507869	3658	-1.18	0.24
Logical Memory			
cg21450381	2983	-1.65	0.099
cg12507869	2983	-2.03	0.043
MMSE			
cg21450381	6780	-5.66	**1.6x10** ^**-8**^
cg12507869	6780	-1.26	0.21
Trail-making Test			
cg21450381	2549	1.06	0.29
cg12507869	2549	0.87	0.38
Phonemic Verbal Fluency			
cg21450381	6390	-0.61	0.54
cg12507869	6390	-5.96	**2.5x10** ^**-9**^

*MMSE* Mini-mental state examination

### Variation in results when modifying the significance threshold

Using a less conservative FDR correction for multiple testing identified associations at a *q*-value threshold of 0.05 in both the basic and fully adjusted models for Phonemic Verbal Fluency (*n* = 49 and *n* = 2), MMSE (*n* = 1 for both models), Vocabulary (*n* = 7 and *n* = 3) and Digit Test (*n* = 309 and *n* = 14). The FDR-significant probes are presented in Supplementary Table 4.

**Fig. 2:**
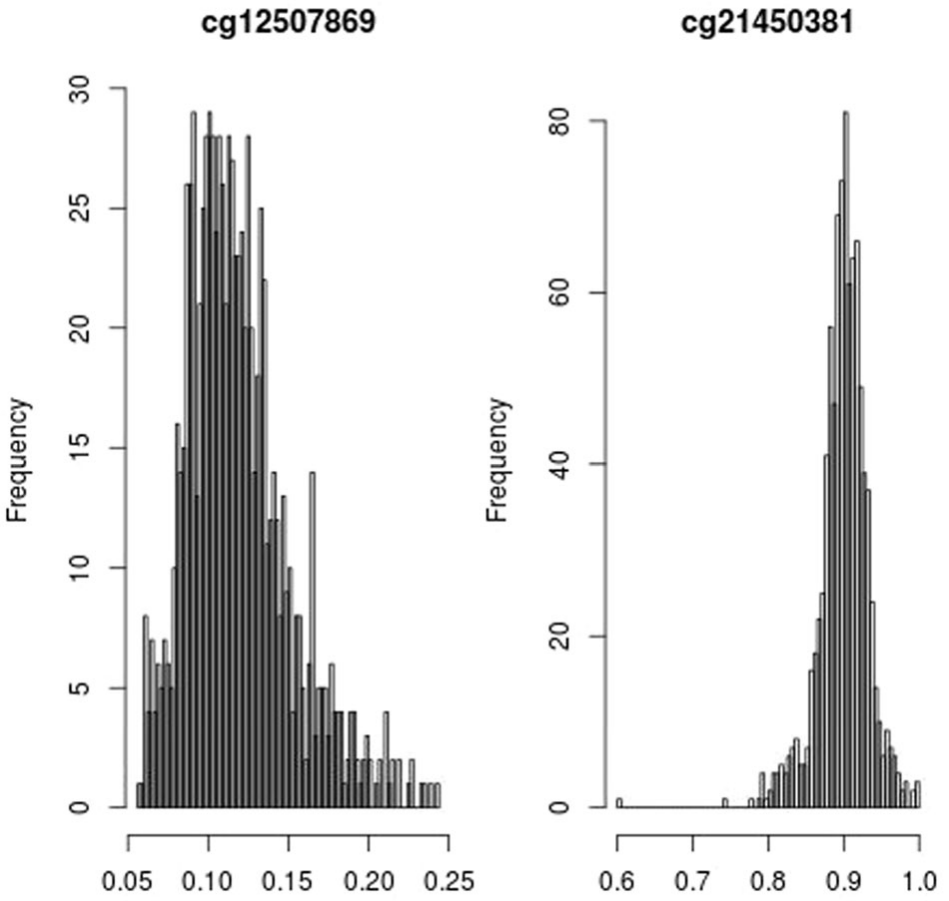
Histogram showing the distribution of beta values for the two significant CpGs in the Lothian Birth Cohort 1936 (*n* = 920)

**Fig. 3:**
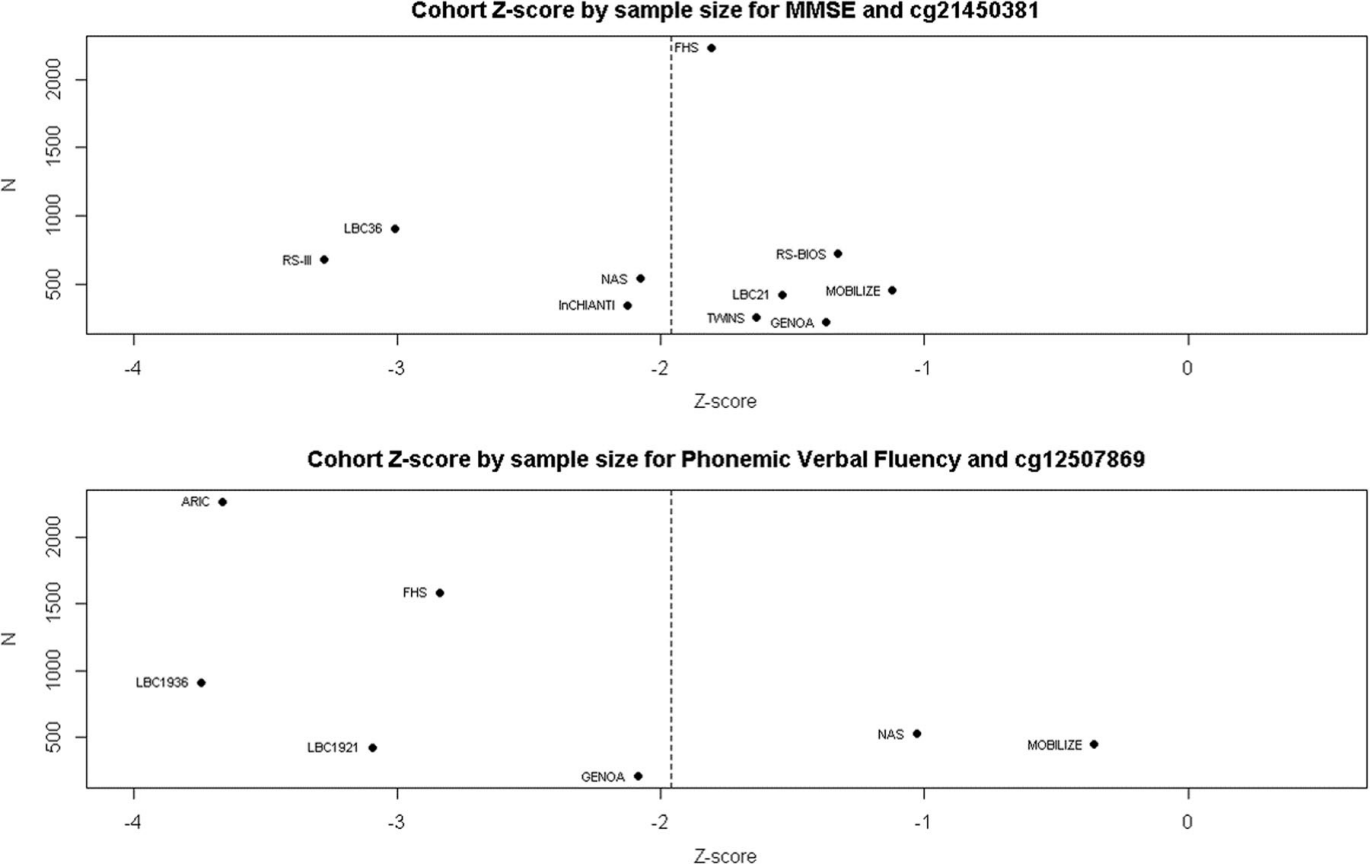
Forest plots of the *Z*-scores by cohort sample size for the two significant CpGs. ARIC Atherosclerosis Risk in the Community, FHS Framingham Heart Study Offspring Cohort, GENOA Genetic Epidemiology Network of Arteriopathy, InCHIANTI Invecchiare in Chianti, LBC Lothian Birth Cohort, MOBILIZE Maintenance of Balance, Independent Living, Intellect and Zest in the Elderly of Boston, NAS Normative Aging Study, RS Rotterdam Study, RS-Bios Rotterdam Study—Biobank-based Integrative Omics Studies

After Bonferroni correction for CpG sites *and* the seven cognitive traits—*P* < 0.05/(420,000*7)—two remaining differentially methylated CpGs were cg21450381 (*R*^2^ = 0.47%, *P* = 1.6 × 10^-8^) with MMSE scores, and cg12507869 (*R*^2^ = 0.55%, *P* = 2.5 × 10^-9^) with Phonemic Verbal Fluency. In both cases, higher methylation was associated with lower cognitive scores across all of the contributing cohorts. cg21450281 is located in an intergenic region of chromosome 12; cg12507869 is located in the inositol polyphosphate-5-phosphatase, 40 kDa (*INPP5A*) gene on chromosome 10. Both probes were approximately normally distributed in the Lothian Birth Cohort 1936 (Fig. 2). A forest plot of the *Z*-scores by cohort sample-size is presented in Fig. 3 and shows no evidence of ethnic outliers or single cohorts driving the associations.

### Combined meta-analysis of all seven cognitive traits

There was no evidence from the combined meta-analytic results of the seven tests for a globally significant CpG across all tests in the fully adjusted model (minimum Benjamini-Hochberg FDR *q*-value of 0.057 for cg12507869).

### Genetic contributions to cognitive-related differential methylation

A methylation QTL lookup [44] analyses identified no SNPs to be associated with cg21450381. The top SNP for cg12507869 (rs113565688 in the *INPP5A* gene on chromosome 10) explained around 1.2% of the variance in methylation (*P*-values of 3.6 × 10^-13^ and 5.4 × 10^-5^ in the Australian and Scottish cohorts, respectively). There is no overlap of this SNP with cognitive traits based on a recent GWAS conducted in the UK Biobank cohort: rs113565688 association with memory (*P* = 0.55), reaction time (*P* = 0.42), verbal-numerical reasoning (*P* = 0.17) and educational attainment (*P* = 0.13) [8].

### Longitudinal changes in methylation at cognitive-related differential methylation sites

Longitudinal analyses over three waves of data (ages 70, 73 and 76 years) from LBC1936, adjusting for sex, imputed white-blood cell counts and technical variables, found no evidence for a linear change in the methylation of either probe over a relatively narrow period in later-life. The mixed model standardised effect size for change in cg12507869 was 0.02 standard deviations per year, *P* = 0.13; the standardised effect size for cg21450381 was -0.02, *P* = 0.40. Without adjustment for covariates, the across wave correlations for cg12507869 were 0.62 (age 70: age 73), 0.63 (age 70: age 76) and 0.68 (age 73: age 76). The corresponding correlations for cg21450381 were 0.04, 0.10 and 0.30, respectively.

### Association of brain MRI features with cognitive-related differential methylation

There were no significant associations between the top two CpGs and either of the brain MRI measures of white matter connectivity (mean diffusivity minimum *P* = 0.56; fractional anisotropy minimum *P* = 0.28) at age 73 in the LBC1936 (*n* = 552).

### Correlation of blood and brain methylation at the cognitive-related differential methylation

Two blood–brain comparisons were conducted. The first, using a blood–brain DNA methylation comparison tool [18] [http://epigenetics.essex.ac.uk/bloodbrain/], provided no evidence for a significant correlation between blood-methylation at either probe with methylation in four brain regions (prefrontal cortex, entorhinal cortex, superior temporal gyrus and cerebellum, Supplementary Figs. 1 and 2). Whereas the mean of the cg21450381 probe was similar to the means for the four brain regions, the mean of the cg12507869 probe in blood was markedly different (hypomethylated) to the means for the prefrontal cortex, entorhinal cortex, superior temporal gyrus (Supplementary Figs. 1 and 2). It was, however, similar to the mean of the cerebellum. The second comparison, using BECon [45] [https://redgar598.shinyapps.io/BECon/] showed the same mean methylation levels for cg21450381 between blood and Brodmann areas 7, 10 and 20; cg12507869 was again hypomethylated in blood compared to the three brain regions. There were moderate correlations between blood-methylation and Brodmann area 20 for both CpGs (*r* = 0.43 for cg12507869 and *r* = -0.46 for cg21450381) and between Brodmann area 7 and cg12507869 (*r* = 0.31).

### Association of cognitive-related differential methylation with Braak staging and Alzheimer’s disease

None of the six CpGs that were epigenome-wide significant in the fully adjusted EWASs at *P* < 1.2 × 10^-7^ were associated with Braak staging or Alzheimer's case–control status in either blood or brain-based methylation (minimum FDR *q*-value 0.51, Supplementary Table 5).

### Transcriptome-wide association study

There were no significant TWAS results for cg21450381. The minimum *P*-value observed was 0.00013 (*Q* = 0.51). There were nine significant TWAS results for cg12507869 at *P* < 2.8 × 10^-6^ and 41 at *Q* < 0.05. There was a nominal inverse association between the *INPP5A* transcript and CpG (*P* = 0.049, *Q* = 0.65). The full TWAS output for the two CpGs is shown in Supplementary Tables 6 and 7.

## Discussion

This study presents a meta-analysis of the relationship between blood-based DNA methylation and cognitive function. We analysed seven different cognitive tests and found two epigenome-wide methylation correlations: cg21450381, located in an intergenic region of chromosome 12, with global cognitive function (as measured by the MMSE); and cg12507869, located in the *INPP5A* gene on chromosome 10, with phonemic verbal fluency. Methylation at the latter CpG was also associated with two other cognitive tests (logical memory and vocabulary) at a nominal *P* < 0.05 threshold. Genetic analyses of the top two CpGs showed a modest *cis* regulation for one of the probes, suggesting that the vast majority of the methylation variation at the cognitive-related differentially methylated sites are due to environmental influences. Blood-based methylation levels at both of the CpGs correlated with methylation levels in Brodmann area 20 (cerebral cortex).

*INPP5A* is a member of the inositol polyphosphate-5-phosphatase (INPP5) family of genes that encode enzymes that hydrolyse inositol 1,4,5 triphosphate (IP3). It is involved in the mobilisation of intracellular calcium, and has been implicated in cerebellar degeneration in mice [53]. A second INPP5 family member, *INPP5D*, has been associated with Alzheimer’s Disease and cognitive decline [54, 55], further implicating this gene family in cognitive functions. cg21450381 is located in an intergenic region of chromosome 12, that contains a histone modification mark (H3K27Ac), DNAaseI hypersensitivity clusters and evidence of transcription factor-binding sites, which indicates that the region may be involved in gene regulation [56].

In a TWAS analysis of the top two probes in the Framingham Heart Study (*n* > 1900), there was no evidence for an association between cg21450381 and blood-based gene expression. Of the nine Bonferroni-significant transcripts in the TWAS of cg12507869, eight were *trans* associations, with the *cis* association occurring in *ADAM12*, which is more than 6 Mb from *INPP5A*. There was no evidence of a *cis* effect of the CpG on the *INPP5A* expression levels.

Disentangling correlation from causation is particularly tricky when studying epigenetic marks in a non-target tissue. By increasing the sample sizes of the meta-analytic EWAS and replicating any findings across different cognitive domains will reduce the chances of false-positive associations. It is, of course, possible that a reliable blood-based epigenetic marker of cognitive function may be several degrees of separation away from the biological processes that drive cognitive skills. For example, the signal could be in response to neurotoxic events, such as inflammation, oxidative stress or small vessel disease. However, the discrimination of cause from consequence is something that affects many epigenetic epidemiology studies. Approaches that may overcome this include Mendelian randomisation studies where a methQTL can be used as an instrument, or the use of mouse models to dissect functional consequences of DNA methylation on gene regulation.

There are additional limitations of this study: a varying number of participants with cognitive data available for each test; heterogeneity in relation to the ethnicity and geographical location of the participants across cohorts; and relating a blood-based methylation signature to a brain-based outcome. We attempted to counter these limitations by: plotting cohort sample-size by *Z*-score to see if there was bias due to outliers or clustering by ethnicity; adjusting for population stratification in the cohorts with admixture; correlating the blood-based CpG associations with methylation levels in several brain regions; looking at the association between brain region-specific methylation and Alzheimer's disease phenotypes for the blood-based CpG associations. It is possible that bias may have been introduced in the secondary analyses that focussed on the MRI, gene expression and longitudinal methylation data, as both the LBC1936 and Framingham studies contributed to the discovery meta-analyses. Re-running the meta-analyses without these cohorts yielded: *P*-values of 1.3 × 10^-7^ and 7.1 × 10^-6^ for the phonemic verbal fluency finding (cg12507869), excluding Framingham and LBC1936, respectively; and *P*-values of 1.7 × 10^-8^ and 3.3 × 10^-6^ for the MMSE finding (cg21450381), again excluding Framingham and LBC1936, respectively. Whereas the longitudinal methylation and MRI findings were null, the *cis* and *trans* expression-methylation associations warrant confirmation in an independent sample. The methQTL findings were based on highly stringent discovery and replication *P*-value thresholds in both LBC and an independent cohort, BSGS.

Neither of the top two CpGs showed signs of linear change in methylation levels between the ages of 70 and 76 years in one of the participating studies (LBC1936) that had three waves of longitudinal data. It is possible that non-linear changes may be present although additional waves of data collection would be required to test this robustly. In addition, a 6-year window is possibly too narrow to observe substantial changes in the CpG levels.

It is notable that the two significant CpG associations were found for the cognitive tests that were completed by the largest number of participants (*n* > 6000). The study provided results for a list of cognitive tests that cover several major cognitive domains: memory, processing speed, executive function, vocabulary and global ability. The heterogeneity with respect to ethnicity and geographic location can allows us to generalise our findings to multiple populations.

Blood is the most feasible tissue for epigenetic epidemiology analyses of cognitive function. Brain would be the ideal target tissue although this would make it impossible to have simultaneous cognitive function data. Moreover, epigenome-wide studies of other brain-related outcomes, such as schizophrenia, have identified putative blood-based methylation signatures [22].

In conclusion, we have presented evidence for blood-based epigenetic correlates of cognitive function. Specifically, we identified methylation sites that are linked to an aspect of executive function and global cognitive ability. The latter finding relied on a relatively crude cognitive test (the MMSE), which is commonly used to identify individuals at risk of dementia. One of the two CpG sites identified was under modest genetic control, with a *cis* SNP explaining over 1% of its variance. Unlike other traits, such as smoking and body mass index [15, 17], there are relatively modest methylation signatures for cognitive function. However, our analyses concur with other recent studies to suggest that blood-based methylation signatures may be useful tools to interrogate differences in brain-related outcomes.

## Electronic supplementary material

Appendix

Supplementary Tables

Supplementary Figure 1

Supplementary Figure 2
