# Supplementary material for: Meta-analysis of epigenome-wide association studies of cognitive abilities
Source: Mol Psychiatry. 2018 Jan 8;23(11):2133–44. doi: 10.1038/s41380-017-0008-y (PMC6035894; doi:10.1038/s41380-017-0008-y)
Supplement: Supplementary file 1 — Appendix [file 41380_2017_8_MOESM1_ESM.docx]

**Overview**

This document summarises the individual cohorts that contributed to the meta-analysis including: cohort descriptions, ethics details, cognitive test and covariate measurement details, and DNA methylation quality control steps. All cohorts assessed DNA methylation using the Infinium HumanMethylation450 BeadChip (Illumina Inc, San Diego, CA)

**Atherosclerosis Risk in Communities (ARIC) Study:**

*Cohort summary*

The ARIC Study is a prospective longitudinal investigation of the development of atherosclerosis and its clinical sequelae in which 15,792 individuals aged 45 to 64 years were enrolled at baseline. A detailed description of the ARIC study has been reported previously.^1^ At the inception of the study in 1987-1989, participants were selected by probability sampling from four communities in the United States: Forsyth County, North Carolina; Jackson, Mississippi (African-Americans only); the suburbs of Minneapolis, Minnesota; and Washington County, Maryland. Four examinations were carried out at three-year intervals (exam 1, 1987-1989; exam 2, 1990-1992; exam 3, 1993-1995; exam 4, 1996-1998.) A fifth clinical examination was completed in 2011-2013. Subjects were contacted annually to update their medical histories between examinations.

*Ethics*

Written informed consent was provided by all study participants, and the study design and methods were approved by institutional review boards at the collaborating medical institutions: University of Mississippi Medical Center Institutional Review Board (Jackson Field Center); Wake Forest University Health Sciences Institutional Review Board (Forsyth County Field Center); University of Minnesota Institutional Review Board (Minnesota Field Center); and the Johns Hopkins School of Public Health Institutional Review Board (Washington County Field Center).

*Cognitive testing*

Cognitive testing was performed in the entire cohort at exams 2, 4, and 5, and was also assessed in subsets of participants at exam 3 (Forsyth County and Jackson only) and in two ancillary studies of carotid magnetic resonance imaging and brain magnetic resonance imaging.^2-4^ Cognitive function was assessed by three neuropsychological tests at each of these examinations and has been described previously.^5^ Two of these were included in this study: 1) the Digit Symbol Substitution Test, a subtest of the Wechsler Adult Intelligence Scale-Revised, tests processing speed and requires timed translation of number to symbols using a key. The score is calculated as the number of correct translations within 90 seconds;^6^ and 2) the Word Fluency Test, a measure of executive function. In three separate 1-minute trials, the participant is asked to generate as many words as possible beginning with the letters F, A, and S. The score is the combined total of correct words produced.^7,8^ DNA methylation data obtained at the same exam as the tests of cognitive function was available for 2,304 African-American study participants with Digit Symbol Substitution Test scores, and for 2,307 participants with Word Fluency Test scores. Individuals were excluded from the analysis if they had a history of prevalent or unknown history of stroke at exam 2 or exam 3.

*DNA methylation*

Detailed information on the DNA methylation assay, quality control procedures, and data normalization have been reported previously.^9^ In brief, bisulfite converted DNA extracted from peripheral blood leukocytes was hybridized to the Illumina HumanMethylation450 BeadChip following the Illumina HD Methylation protocol (Illumina Inc., San Diego, CA). An average detection p-value <0.01 was used as a measure of performance for each probe and was determined using an algorithm in the GenomeStudio® software module (Illumina, Inc.) to assess whether its methylation level was above background using negative control bead types included on the array. Individuals were excluded from the analyses if the pass rate for the DNA sample for the participant was < 99% (probes with a detection p-value <0.01/all probes on the array). At the target level, poor-quality CpG sites with average detection p-value > 0.01 were flagged, and the percentage of samples having detection p-value >0.01 for each autosomal and X chromosome CpG site was calculated. CpG sites were not analyzed in this study if more than 5% of the samples showed a detection p-value > 0.01, or if the average detection p-value was > 0.01 on the Y chromosome for males. Since there are known technical differences between the two probe designs (type I and type II) present on the array, methylation values were normalized using the Beta MIxture Quantile dilation (BMIQ) method.^10^

*Analysis*

Surrogate variables were constructed to control batch effects.^11^ Since the various blood subpopulations have different methylation patterns and can confound DNA methylation association studies,^12^ the proportions of neutrophils, lymphocytes, monocytes, eosinophils, and basophils were imputed using the Houseman method^13^ based on the measured differential cell counts available for a subset of ARIC participants at exam 2 (n = 175). All association analyses were performed in R using linear mixed models with the DNA methylation beta value for each probe as the outcome variable.

*Covariates*

The primary regression model was adjusted for cognitive score, standardized age, sex, field center, exam, white blood cell proportions, technical variables (plate number, chip row number, chip column number) and 5 principal components from the Illumina Infinium HumanExome BeadChip genotype array (Illumina, Inc.) to account for potential confounding by genetic ancestry. A second regression model included the variables in the primary model and was further adjusted for age^2^, an interaction term for age by gender, smoking status, and body mass index (BMI). BMI was calculated as weight in kilograms/(height in meters)^2^ from height and weight measurements obtained at the same examination as the DNA methylation data. Similarly, information on cigarette smoking was obtained at either exam 2 or 3 using an interviewer-administered questionnaire and was classified as current, former, or never.

*Acknowledgements*

The Atherosclerosis Risk in Communities (ARIC) study is carried out as a collaborative study supported by the National Heart, Lung, and Blood Institute (NHLBI) contracts (HHSN268201100005C, HHSN268201100006C, HHSN268201100007C, HHSN268201100008C, HHSN268201100009C, HHSN268201100010C, HHSN268201100011C, and HHSN268201100012C). The authors thank the staff and participants of the ARIC study for their important contributions. Funding support for “Building on GWAS for NHLBI-diseases: the U.S. CHARGE consortium” was provided by the NIH through the American Recovery and Reinvestment Act of 2009 (ARRA) (5RC2HL102419).

**Framingham Heart Study (FHS) Offspring Cohort**

*Cohort summary*

The FHS is a single-site, community-based, ongoing cohort study that was initiated in 1948 to investigate prospectively the risk factors for CVD. The Original cohort (n=5209) were randomly recruited in the town of Framingham, MA, USA, and examined every 2 year since 1948;^14^ their children and spouses of the children (n=5124), the Offspring cohort, were enrolled in 1971 and examined approximately every 4 year since 1971.^15^ DNA methylation was measured on 2,846 Offspring participants around 8^th^ examination cycle (2005-2008). This study sample of Offspring individuals with cognitive measures and methylation ranges from 2,356 (MMSE) to ~1700 (other cognitive measures).

*Ethics*

Written informed consent to genetic research has been obtained on all individuals included in this study. Ethics permission for FHS and genetic research in FHS was obtained from the Institutional Review Board of Boston University Medical Campus (IRB number H-32132, H-26671)

*Cognitive testing*

The Mini-Mental State Examination (MMSE)^16^ was assessed in FHS Offspring cohort at 7^th^ examination cycle with participants scoring lower than 24 excluded from this study.

The rest of cognitive measures were assessed between 2004 and 2011. The measurements obtained closest to the mid of examination 8 cycle (5/19/2007) were used in this study.

Logical Memory was assessed using the Wechsler Memory Scale III-UK.^17^ A paragraph was read aloud and the participant was asked to recall its content, immediately and after a delay. There are 25 items in the story, yielding a sum score range of 0-50.

Semantic fluency (Animal naming) and phonemic fluency (FAS) from the Controlled Oral Word Association Test (COWAT^18^) was assessed in FHS. The number of animal names generated in 1 minute and the number of words generated in 1 minute beginning with the letters F, A, or S were obtained respectively.

Vocabulary was measured in FHS using the Wide Range Achievement Test III edition (WRAT-III^19^), which is an achievement test for accessing an individual's ability to read words, comprehend sentences, spell, and compute solutions to math problems

Attention and executive function is assessed by Trail Making Test Part A and B.^20^ Participants were administrated a sheet of paper with 25 circles in both parts of the Trail Making Test. In Part A, the circles are numbered 1 – 25, and the participant was instructed to draw lines to connect the numbers in ascending order. In Part B, the circles include both numbers (1 – 13) and letters (A – L); as in Part A, the participant draws lines to connect the circles in an ascending pattern, but with the added requirement of alternating between the numbers and letters (i.e., 1-A-2-B-3-C, etc.).

*Methylation*

During the 8^th^ examination cycle (2005-2008), peripheral whole blood samples were collected from 2,846 Offspring participants, buffy coat fractions were obtained and genomic DNA was extracted using the Gentra Puregene DNA extraction kit (Qiagen, , Venlo, Netherlands). Bisulfite conversion of genomic DNA was performed with the EZ DNA Methylation Kit (Zymo Research, Irvine, CA). After whole genome amplification, fragmentation, array hybridization, and single-base pair extension, DNA methylation was quantified in two laboratories. The first laboratory analyzed 576 samples that were previously selected for a cardiovascular disease (CVD) case-control study.^21^ The second laboratory analyzed 2, 270 samples from the remainder of the Offspring cohort.

*Covariates*

Current smoking is defined as regularly smoking in the past year which was self-reported at each examination. Using examination 8 and previous examination data, participants were classified as current smoker, ex-smoker, never smoker. Body mass index was measured as weight in kilograms divided by height (in metres squared).

*Gene Expression*

Total RNA was isolated from frozen PAXgene blood tubes (PreAnalytiX, Hombrechtikon, Switzerland) and amplified using the WT-Ovation Pico RNA Amplification System (NuGEN, San Carlos, CA). The obtained cDNA was hybridized to the Affymetrix Human Exon 1.0 ST Array (Affymetrix, Inc., Santa Clara, CA). The raw data were quantile-normalized and log2 transformed, followed by summarization using Robust Multi-array Average. The gene annotations were obtained from Affymetrix NetAffx Analysis Center (version 31). Transcript clusters that were not mapped to RefSeq transcripts were excluded, resulting in 17,873 distinct transcripts (17,324 distinct genes). A partial least square method was used to impute white blood cell and platelet counts and the percentage of lymphocytes, monocytes, eosinophils and basophils from gene expression data on measured cell counts in 2284 participants from FHS Second and Third Generation Cohorts. The percentages of each imputed cell type were then normalized, where the negative predicted values were set to 0 and the sum of the percentages for all cell types were set 100%.

*Acknowledgements*

We thank the study participants, as well as the study team (especially the investigators and staff of the neurology core) for their contributions. This work was supported by the National Heart, Lung and Blood Institute’s Framingham Heart Study Contract No. N01-HC-25195 and No. HHSN268201500001I, and by grants from the National Institute of Aging (R01s AG033193, AG008122, AG054076, AG033040, AG049607, AG05U01-AG049505), and the National Heart, Lung and Blood Institute (R01 HL093029, HL096917). The laboratory work for this investigation was funded by the Division of Intramural Research, National Heart, Lung, and Blood Institute, National Institutes of Health, and by NIH contract N01-HC-25195. The analytical component of this project was funded by the Division of Intramural Research, National Heart, Lung, and Blood Institute, and the Center for Information Technology, National Institutes of Health.

**Genetic Epidemiology Network of Arteriopathy (GENOA)**

*Cohort summary*

The Genetic Epidemiology Network of Arteriopathy (GENOA) study is a community-based study of hypertensive sibships that was designed to investigate the genetics of hypertension and target organ damage in African Americans from Jackson, Mississippi and non-Hispanic whites from Rochester, Minnesota.^22^ In the initial phase of the GENOA study (Phase I: 1996-2001), all members of sibships containing ≥ 2 individuals with essential hypertension clinically diagnosed before age 60 were invited to participate, including both hypertensive and normotensive siblings. Exclusion criteria of the GENOA study were secondary hypertension, alcoholism or drug abuse, pregnancy, insulin-dependent diabetes mellitus, or active malignancy. Eighty percent of African Americans (1,482 subjects) and 75% of non-Hispanic whites (1,213 subjects) from the initial study population returned for the second examination (Phase II: 2001-2005). Study visits were made in the morning after an overnight fast of at least eight hours. Demographic information, medical history, clinical characteristics, lifestyle factors, and blood samples were collected in each phase. DNA methylation levels were measured only in African Americans participants. After quality control, DNA methylation and cognitive data were available for 219 African Americans.

*Ethics*

Written informed consent was obtained from all subjects, and approval was granted by participating institutional review boards at the University of Mississippi Medical Center, the Mayo Clinic, and the University of Michigan.

*Cognitive testing*

The Mini-Mental State Examination (MMSE)^16^ was assessed, and participants scoring lower than 24 were excluded from the EWAS.

Animal naming^7^ was assessed as the number of animals that a participant could name in one minute.

Verbal fluency^7^ was assessed as the sum score from three trials. The participant was asked to name as many words as possible that begin with F in one minute. This was repeated for words beginning with A and S.

The Trailmaking Test^7^ was assessed as the number of time it took to complete the trail containing both letters and numbers (Trailmaking Test Part B). The total time was truncated at 360 seconds, and participants who made more than 5 errors were assigned the maximum time value (360). The variable was transformed using the natural log prior to analysis.

Digit symbol coding was assessed using the Wechsler Adult Intelligence Scale Revised (WAIS-R^23^). The participant had to recode as many number-symbol codes as possible in 90 seconds.

*Methylation*

Genomic DNA of 422 participants were extracted from stored peripheral blood leukocytes, bisulfite converted, and then measured for DNA methylation using the Illumina Infinium HumanMethylation450 BeadChip using stored blood samples collected during the Phase I examination. The Minfi R package^24^ was used to preprocess, normalize (using *SWAN*)^25^, and calculate beta values. The proportion of each cell type were estimated using Houseman’s method.^13^ Detection p-value for each site was calculated for each person. For the sites with detection p-value>0.01, the corresponding beta value was set as missing. All samples had >95% of probes with p-value <0.01. A total of 1707 probes were removed because >5% of samples had a detection p-value >0.01.

*Covariates*

Self-reported smoking status was categorized into three groups: current smoker (smokes currently or quit smoking within the past year), ex-smoker (formerly smoked, but quit more than 1 year ago), and never-smoker (smoked less than 100 cigarettes in his/her entire life). Body mass index was measured as weight in kilograms divided by height (in meters squared).

*Acknowledgements*

Support for the Genetic Epidemiology Network of Arteriopathy (GENOA) was provided by the NHLBI (HL054457, HL100185, HL119443, and HL133221) and the NINDS (NS041558) of the NIH.

**InCHIANTI (Invecchiare in Chanti)**

*Cohort summary*

InCHIANTI is a population-based prospective cohort study of residents from two areas in the Chianti region of Tuscany, Italy. Study participants were enrolled between 1998 and 2000 and were followed at 3-year intervals for 9 years. Selection of study participants and data collection procedures have been previously described.^26^ Overall, 1,326 participants donated a blood sample at baseline (1998–2000) and, of these, 784 also donated a blood sample at the 9-year follow-up (2007–2009). Genome-wide DNAm was assayed on DNA samples corresponding to participants with sufficient DNA at both visits. The study population for the present analysis includes individuals with DNAm data at baseline meeting quality control criteria, excluding any individuals who had suffered a stroke or with dementia (MMSE < 24).

*Ethics*

InCHIANTI protocols were approved by the Instituto Nazionale Riposo e Cura Anziani institutional review board in Italy and study participants provided informed consent.

*Cognitive testing*

Trained interviewers administered the MMSE and the Trail Making Test.

The Mini-Mental State Examination (MMSE)^16^ was assessed in the InChinati cohort with participants scoring lower than 24 excluded from the EWAS.

The Trail Making Test part B was assessed in the InChianti cohort; time to complete the task was log transformed prior to analysis and outliers more than 3.5 standard deviations from the mean were Winsorized.

*Methylation*

Detailed information about the collection and QC steps undertaken on the InChianti methylation data have been reported previously^27^ Briefly, initial data analysis was performed using GenomeStudio 2011.1 (Model M Version 1.9.0, Illumina Inc.). Threshold call rate for inclusion of samples was 95%. Quality control of sample handling included comparison of clinically reported sex versus sex of the same samples determined by analysis of methylation levels of CpG sites on the X chromosome.^28^ Quality filtering and normalization was performed using the DASEN method in the R package “watermelon”.^29^ Markers were removed if the bead count was less than 3 in ≥5% of samples. Samples and markers were also excluded if ≥5% of detection p values were greater than .01. A background adjustment and quantile normalization were applied to the filtered data set; the selected method normalizes both methylated and unmethylated probes as well as type I and II assays (the 450k array includes both paired probe and single probe assay designs) separately.

*Covariates*

Smoking status was self-reported in three categories: current smoker, ex-smoker, never smoker. Body mass index was measured as weight in kilograms divided by height (in metres squared). White blood cell differential count was assessed on ethylenediaminetetraacetic acid anticoagulated whole blood using a Coulter Counter (LH 750 Hematology Autoanalyzer, Beckman Coulter Inc., Brea, CA) and expressed as percentages of neutrophils, lymphocytes, monocytes, eosinophils, and basophils. Methylation array batch, Sentrix ID and recruitment site were included as technical batch variables.

*Acknowledgements*

The InCHIANTI study baseline (1998–2000) was supported as a “targeted project” (ICS110.1/RF97.71) by the Italian Ministry of Health and in part by the U.S. National Institute on Aging (Contracts: 263 MD 9164 and 263 MD 821336); the InCHIANTI Follow-up 1 (2001–2003) was funded by the U.S. National Institute on Aging (Contracts: N.1-AG-1-1 and N.1-AG-1-2111); the InCHIANTI Follow-ups 2 and 3 studies (2004–2010) were financed by the U.S.

**Lothian Birth Cohorts 1921 and 1936**

*Cohort summary*

The Lothian Birth Cohorts of 1921 and 1936 (LBC1921 and LBC1936) are two longitudinal studies of ageing.^30-32^ They derive from the Scottish Mental Surveys of 1932 and 1947, respectively, when nearly all 11 year old children in Scotland completed a test of general cognitive ability.^30^ Survivors living in the Lothian area of Scotland were recruited in late-life at mean age 79 for LBC1921 (n=550) and mean age 70 for LBC1936 (n=1,091). Follow-up has taken place at ages 70, 73, and 76 in LBC1936 and ages 79, 83, 87, and 90 in LBC1921. Collected data include genetic information, longitudinal epigenetic information, longitudinal brain imaging (LBC1936), and numerous blood biomarkers, anthropomorphic and lifestyle measures. Post QC, DNA methylation data were available for 920 LBC1936 participants at age 70 and for 446 LBC1921 participants at age 79.

*Ethics*

Following written informed consent, venesected whole blood was collected for DNA extraction in both LBC1921 and LBC1936. Ethics permission for the LBC1921 was obtained from the Lothian Research Ethics Committee (Wave 1: LREC/1998/4/183). Ethics permission for the LBC1936 was obtained from the Multi-Centre Research Ethics Committee for Scotland (Wave 1: MREC/01/0/56), the Lothian Research Ethics Committee (Wave 1: LREC/2003/2/29), and the Scotland A Research Ethics Committee (Waves 2 and 3: 07/MRE00/58).

*Cognitive testing*

The Mini-Mental State Examination (MMSE)^16^ was assessed in both LBC cohorts with participants scoring lower than 24 excluded from the EWAS. Logical Memory was assessed using Wechsler Memory Scales.^17,33^ A paragraph was read aloud and the participant was asked to recall its content, immediately and after a delay. There are 25 items in the story, yielding a sum score range of 0-50. Verbal fluency^34^ was assessed in both cohorts as the sum score from three trials. The participant was asked to name as many words beginning with C in one minute. This is repeated for words beginning with F and L. Vocabulary was measured using the National Adult Reading Test (NART),^35^ which requires the pronunciation of 50 irregular words (scoring range: 0-50). Digit symbol coding was assessed in LBC1936 using the Wechsler Adult Intelligence Scale III-UK.^36^ The participant had to recode as many number-symbol codes as possible in two minutes.

*Methylation*

Detailed information about the collection and QC steps undertaken on the LBC methylation data have been reported previously.^37^ Briefly, background correction was performed and quality control was used to remove probes with a low detection rate (P>0.01 for >5% of samples), low quality (manual inspection), low call rate (P<0.01 for <95% of probes), and samples with a poor match between genotypes and SNP control probes, and incorrect predicted sex.

*Covariates*

Smoking status was self-reported in three categories: current smoker, ex-smoker, never smoker. Body mass index was measured as weight in kilograms divided by height (in metres squared).

*Acknowledgements*

We thank the cohort participants and team members who contributed to these studies. Phenotype collection in the Lothian Birth Cohort 1921 was supported by the UK’s Biotechnology and Biological Sciences Research Council (BBSRC), The Royal Society and The Chief Scientist Office of the Scottish Government. Phenotype collection in the Lothian Birth Cohort 1936 was supported by Age UK (The Disconnected Mind project). Methylation typing was supported by Centre for Cognitive Ageing and Cognitive Epidemiology (Pilot Fund award), Age UK, The Wellcome Trust Institutional Strategic Support Fund, The University of Edinburgh, and The University of Queensland. REM, SEH, JMS, and IJD are members of the University of Edinburgh Centre for Cognitive Ageing and Cognitive Epidemiology (CCACE), which is supported by funding from the BBSRC, the Medical Research Council (MRC), and the University of Edinburgh as part of the cross-council Lifelong Health and Wellbeing initiative (MR/K026992/1).

**MOBILIZE Cohort**

*Cohort summary*

The MOBILIZE Boston Study (MBS) stands for "Maintenance of Balance, Independent Living, Intellect, and Zest in the Elderly of Boston". This cohort study is part of the Hebrew Rehabilitation Center/Harvard Research Nursing Home Program Project.^38^ It was designed to target novel risk factors for falls, and included analysis for pain, cerebral hypoperfusion, and foot disorders in the older population. This study is based in the Institute for Aging Research (IFAR) at Hebrew SeniorLife, a large geriatric housing, health care, and research organization. Using a door-to-door population-based recruitment, the study enrolled 765 persons aged 70 and older. The participants are largely representative of seniors in the Boston area in terms of age, sex, race and Hispanic ethnicity. The average age of study participants was 77.9 years (s.d. 5.5) and nearly two-thirds were women. The study cohort was 78% white and 17% black.

*Ethics*

The MOBILIZE Boston Study was approved by the Institutional Review Boards of Hebrew SeniorLife and the collaborating institutions.

*Cognitive testing*

Verbal fluency was assessed with phonemic and semantic fluency tasks.^39^ Other cognitive assessment included The Trailmaking Test (parts A and B), as a measure of executive function, is frequently used in the clinical setting and has been shown to be sensitive to the presence of frontal lobe pathology.^40^ Other cognitive functions evaluated in MOBILIZE Boston Study included: verbal memory functioning, using the Hopkins Verbal Learning Test – Revised (HVLT-R) and a cognitive screening measure, using the the Clock-in-a-Box Test (CIB), a modification of the commonly used Clock Drawing test.^41^

*Methylation*

DNA was extracted from buffy coat using the QIAamp DNA Blood Kit (QIAGEN, Valencia, CA). 500 ng of DNA was used to perform bisulfite conversion using the EZ-96 DNA Methylation Kit (Zymo Research, Orange, CA). To reduce the chip and plate effects, we used a two-stage age-stratified algorithm to randomise samples and ensure similar age distributions across chips and plates; 12 samples – which were sampled across all the age quartiles – were randomized to each chip, then chips were randomised to plates (each using eight chips). Quality control analysis was performed to remove samples and probes, where >1% of probes or samples, respectively, had a detection p-value > 0.05. The remaining samples were preprocessed using the Illumina-type background correction and normalized with the dye-bias and BMIQ adjustments and between array normalization which were used to generate beta methylation values. 484,411 CpG probes were in the working set.

*Covariates*

Data about smoking status was reported by in three categories: never smoker, former smoker, and current never smoker. Body mass index was determined in kilograms by metres squared, using the weight in kilograms divided by squared-height.

*Acknowledgements*

We acknowledge the MOBILIZE Boston Study (HRCA/Harvard Research Nursing Home Project, grant number AG04390).

**Normative Aging Study (NAS)**

*Cohort*

The US Department of Veterans Affairs (VA) Normative Aging Study (NAS) is an ongoing longitudinal cohort of aging men established in 1963. Participants were 21-80 years of age and free of known chronic medical conditions at enrolment.^42^ Clinical health data and demographic factors are collected during medical exams at 3-5 year intervals and supplemented with behavioural data obtained from periodic mail surveys. In the period 1999-2007, DNA samples were collected from the 675 active participants. We excluded participants who were non-whites (N=18), with leukaemia (N=3), with a stroke before the visit (N=43), with reported dementia (N=2), and with lobectomy (N=3).

*Ethics*

The NAS study was approved by the Institutional Review Board (IRB) of VA Boston Healthcare System. Participants have provided written informed consent at each visit.

*Cognitive testing*

Starting in 1993, all participants reporting for in-person visits underwent cognitive testing.^43^ In this study we considered two cognitive tests: the Mini-Mental State Examination (MMSE) and the semantic verbal fluency task (animal naming).

The MMSE is a validated global cognitive test to screen for dementia.^44^ MMSE assesses several cognitive domains, such as orientation, immediate and short-term recall, attention and calculation, word finding, construction reading and writing skills, and ability to follow a three-step command. In this study the maximum possible score on the MMSE was 29, because the question on the county of residence was excluded as counties in Massachusetts have little political meaning and are generally not known and, thus, not of diagnostic utility.^43^ Participants with MMSE scores lower than 24 excluded, leaving a total of 538 participants for the corresponding analysis.

The Verbal Fluency task assesses language (vocabulary size, naming), response speed, mental organization, search strategies, short- and long-term memory, letting each participant say as many words as possible from a category in a given time. Participants with missing information on verbal fluency test were excluded from the corresponding analysis, leaving a total of 528 participants. We Winsorised a single outlier of verbal fluency test.

*Methylation*

DNA was extracted from buffy coat using the QIAamp DNA Blood Kit (QIAGEN, Valencia, CA). 500 ng of DNA was used to perform bisulfite conversion using the EZ-96 DNA Methylation Kit (Zymo Research, Orange, CA). To reduce the chip and plate effects, we used a two-stage age-stratified algorithm to randomize samples and ensure similar age distributions across chips and plates; 12 samples – which were sampled across all the age quartiles – were randomized to each chip, then chips were randomized to plates (each housing eight chips). Quality control analysis was performed to remove samples and probes where >1% of probes and samples, respectively, had a detection p-value > 0.05. The remaining samples were preprocessed using the Illumina-type background correction^45^ and normalized with the dye-bias and BMIQ^46^ adjustments, which were used to generate beta methylation values. 477,928 CpG probes were in the working set.

*Covariates*

At each in-person examination visit, participants provided demographic, lifestyle and anthropometric information, e.g. age (continuous), self-reported smoking status (never/former/current), and Body Mass Index (BMI) (continuous – Kg/m2). Anthropometric measures were performed with participants in undershorts and socks. Waist circumference was measured in centimetres at the umbilical level, perpendicular to the axis of the upper body. White blood cell measures were estimated using the gold standard rule.^13^

*Acknowledgments*

The present work on the US Department of Veterans Affairs (VA) Normative Aging Study has been supported by funding from the U.S. National Institute of Environmental Health Sciences (NIEHS) (R01ES025225, P30ES009089, R01ES015172, R01ES021733). The VA Normative Aging Study is supported by the Cooperative Studies Program/ Epidemiology Research and Information Center ERIC, US Department of Veterans Affairs, and is a research component of the Massachusetts Veterans Epidemiology Research and Information Center (MAVERIC), Boston, MA. Additional support to the VA Normative Aging Study was provided by the US Department of Agriculture, Agricultural Research Service (contract 53-K06-510). The views expressed in this paper are those of the authors and do not necessarily represent the views of the US Department of Veterans Affairs.

**Rotterdam study**

*Cohort summary*

The Rotterdam study is a prospective, population-based cohort study that includes inhabitants of a well-defined Ommord district in the city of Rotterdam in the Netherlands.^47^ The cohort was initially defined in 1990 among approximately 7,900 persons, aged 55 years and older, who underwent a home interview and extensive physical examination at the baseline and during the follow-up rounds every 3-4 years (RS-I). The cohort was extended in 2000/2001 (RS-II, 3,011 individuals, aged 55 years and older) and 2006/2008 (RS-III, 3,932 individuals aged 45 and older).^47^ Collected data include genetic information, epigenetic information, imaging (of heart, blood vessels, eyes, skeleton and brain) and numerous blood biomarkers, anthropomorphic and lifestyle measures.^47^ Post QC, DNA methylation and phenotypic data used for the analyses were available for two independent RS datasets including 689 participants from the first visit of RS-III and 728 participants from the third visit of RS-II and the second visit of RS-III. The latter dataset is part of the Biobanking and Biomolecular Resources Research Infrastructure for The Netherlands (BBMRI-NL), BIOS (Biobank-based Integrative Omics Studies) project [<http://www.bbmri.nl/?p=259>].

*Ethics*

All participants provided written informed consent to participate in the study and to obtain information from their treating physicians. The Rotterdam Study has been approved by the Medical Ethics Committee of the Erasmus MC and by the Ministry of Health, Welfare and Sport of the Netherlands , implementing the Wet Bevolkingsonderzoek: ERGO (Population Studies Act: Rotterdam Study).^47^

*Cognitive testing*

The Mini-Metal State Examination (MMSE) was assessed in the RS cohorts.^48^ Participants scoring lower than 24 were excluded from the EWAS.

Letter Digit Substitution Test (LDST)^48,49^ was assessed by a task in which participant were required to assign the number to corresponding letters. The score was defined as the total number of correct combinations completed in one minute.

Verbal fluency^48,49^ was assessed by a task in which participants had to mention as many animals as possible within one minute. The score was defined as the total number of unique correctly named animals.

*Methylation*

Detailed information about the collection and QC steps undertaken on the RS methylation data have been reported previously.^50^ Briefly, quality control was used to remove probes with a detection P-value > 0.01 in 1% of samples, and samples with low call rate (<99%), incomplete bisulfite conversion and gender clustering.

*Covariates*

Smoking status was self-reported in three categories: current smoker, ex-smoker, never smoker. Body mass index was measured as weight in kilograms divided by height (in metres squared).

*Acknowledgements*

The authors are grateful to the Rotterdam Study participants, the staff involved with the Rotterdam Study and the participating general practitioners and pharmacists. The generation and management of the Illumina 450K methylation array data (EWAS data) for the Rotterdam Study was executed by the Human Genotyping Facility of the Genetic Laboratory of the Department of Internal Medicine, Erasmus MC, the Netherlands. The EWAS data was funded by the Genetic Laboratory of the Department of Internal Medicine, Erasmus MC, and by the Netherlands Organization for Scientific Research (NWO; project number 184021007) and made available as a Rainbow Project (RP3; BIOS) of the Biobanking and Biomolecular Research Infrastructure Netherlands (BBMRI-NL). We thank Mr. Michael Verbiest, Ms. Mila Jhamai, Ms. Sarah Higgins, Mr. Marijn Verkerk, and Ms Pooja Madaviya for their help in creating the methylation database.

The Rotterdam Study is funded by Erasmus Medical Center and Erasmus University, Rotterdam, Netherlands Organization for the Health Research and Development (ZonMw), the Research Institute for Diseases in the Elderly (RIDE), the Ministry of Education, Culture and Science, the Ministry for Health, Welfare and Sports, the European Commission (DG XII), and the Municipality of Rotterdam. The authors are grateful to the study participants, the staff from the Rotterdam Study and the participating general practitioners and pharmacists.

**TwinsUK Cohort**

*Cohort summary*

The TwinsUK cohort was established in 1992 and recruited same sex monozygotic and dizygotic twin pairs from the United Kingdom.^51^ There are over 13,000 twin participants in the cohort and the majority of participants are female Caucasians. In the current study we included 332 subjects with DNA methylation data and MMSE and vocabulary measurements obtained during clinical visits.

*Ethics*

All participants provided written informed consent at the time of visits. Ethics for the TwinsUK was obtained from the Guy’s and St Thomas’ (GSTT) Ethics Committee.

*Cognitive testing*

The Mini-Mental State Examination (MMSE) and National Adult Reading Test (NART) were conducted during clinical visits. Participants with MMSE lower than 24 were excluded from the analysis. In total, there were 256 subjects with MMSE records and 118 subjects with NART records.

*Methylation*

Details of the sample collection and experimental process have previously been described.^52^ Raw Illumina methylation beta levels were transformed by the beta mixture quantile dilation (BMIQ) method.^10^ Probes were excluded if they mapped to multiple locations in the reference sequence and if more than 1% of subjects had detection P-value > 0.05 at the probe. Individuals with over 5% missing probes were also excluded.

*Covariates*

We used a linear mixed effects model to adjust for family and zygosity structure as random effects, and all other covariates as fixed effects. The fixed effect covariates included BMI, smoking status (non-smokers, ex-smokers, and current smokers), plate, position on the plate, and four FACS-measured cell counts (lymphocytes, monocytes, neutrophils, and eosinophils).

*Acknowledgements*

We would like to thank all twins and family members in the TwinsUK cohort. Support for this work was obtained from the European Research Council (ERC 250157), from the Economic and Social Research Council (ES/N000404/1), and in part from the TwinsUK resource, which is funded by the Wellcome Trust; the European Community’s Seventh Framework Programme (FP7/2007–2013); and the National Institute for Health Research (NIHR) BioResource, Clinical Research Facility and Biomedical Research Centre based at Guy’s and St Thomas’ NHS Foundation Trust and King’s College London.

**References**

1. The ARIC investigators. The Atherosclerosis Risk in Communities (ARIC) Study: design and objectives. *Am J Epidemiol* 1989; **129**: 687-702

2. Liao D, Cooper L, Cai J, Toole JF, Bryan NR, Hutchinson RG, et al. Presence and severity of cerebral white matter lesions and hypertension, its treatment, and its control. The ARIC Study. Atherosclerosis Risk in Communities Study. *Stroke* 1996; **27**: 2262-70

3. Wagenknecht L, Wasserman B, Chambless L, Coresh J, Folsom A, Mosley T, et al. Correlates of carotid plaque presence and composition as measured by MRI: the Atherosclerosis Risk in Communities Study. *Circ Cardiovasc Imaging* 2009; **2**: 314-22

4. Knopman DS, Mosley TH, Catellier DJ, Coker LH, Atherosclerosis Risk in Communities Study Brain, M.R.I.S. Fourteen-year longitudinal study of vascular risk factors, APOE genotype, and cognition: the ARIC MRI Study. *Alzheimers Dement* 2009; **5**: 207-14

5. Cerhan JR, Folsom AR, Mortimer JA, Shahar E, Knopman DS, McGovern PG, et al. Correlates of cognitive function in middle-aged adults. Atherosclerosis Risk in Communities (ARIC) Study Investigators. *Gerontology* 1998; **44**: 95-105

6. Wechsler D. The Adult Intelligence Scale – Revised. Psychological Corporation, New York, 1981.

7. Lezak M. Neuropsychological Assessment. Oxford University Press, Oxford, 1995.

8. Tombaugh TN, Kozak J, Rees L. Normative data stratified by age and education for two measures of verbal fluency: FAS and animal naming. *Arch Clin Neuropsychol* 1999; **14**: 167-77.

9. Demerath EW, Guan W, Grove ML, Aslibekyan S, Mendelson M, Zhou YH, et al. Epigenome-wide association study (EWAS) of BMI, BMI change and waist circumference in African American adults identifies multiple replicated loci. *Hum Mol Genet* 2015; **24**: 4464-79

10. Teschendorff AE, Marabita F, Lechner M, Bartlett T, Tegner J, Gomez-Cabrero D, et al. A beta-mixture quantile normalization method for correcting probe design bias in Illumina Infinium 450 k DNA methylation data. *Bioinformatics,* 2013; **29**: 189-96.

11. Leek JT, Johnson WE, Parker HS, Jaffe AE, Storey JD. The sva package for removing batch effects and other unwanted variation in high-throughput experiments. *Bioinformatics* 2012; **28**: 882-3

12. Reinius LE, Acevedo N, Joerink M, Pershagen G, Dahlen SE, Greco D et al. Differential DNA methylation in purified human blood cells: implications for cell lineage and studies on disease susceptibility. *PLoS One* 2012; **7**: e41361

13. Houseman EA, Accomando WP, Koestler DC, Christensen BC, Marsit CJ, Nelson HH, et al. DNA methylation arrays as surrogate measures of cell mixture distribution. *BMC bioinformatics* 2012; **13**: 86.

14. Dawber TR, Kannel WB. The Framingham study. An epidemiological approach to

coronary heart disease. *Circulation* 1966; **34**: 553-555.

15. Feinleib M, Kannel WB, Garrison RJ, McNamara PM, Castelli WP. The Framingham Offspring Study. Design and preliminary data. *Prev Med* 1975; **4**: 518-525.

16. Folstein MF, Folstein SE, McHugh PR. Mini-Mental State: A practical method for grading the cognitive state of patients for the clinician. *J Psychiat Res*. 1975, **12**: 189-198.

17. Wechsler D. WMS-IIIUK administration and scoring manual. Psychological Corporation, London, UK, 1998.

18. Benton AL, Hamsher K, Sivan AB. Multilingual Aphasia Examination. 3rd ed AJA Associates; Iowa City, IA: 1994.

19. Wilkinson GS. The Wide Range Achievement Test: Manual (3rd ed.). Wilmington, DE: Wide Range, 1993.

20. Reitan RM. Validity of the trail making test as an indicator of organic brain

damage. *Perceptual and Motor Skills*, 1958; **8**: 271–276.

21. Huan T, Zhang B, Joehanes R, Zhu J, Johnson AD, Ying S. et al. A systems biology framework identifies molecular underpinnings of coronary heart disease. *Arterioscler Thromb Vasc Biol* 2013; **33**: 1427-34

22. Daniels PR, Kardia SL, Hanis CL, Brown CA, Hutchinson R, Boerwinkle E, et al. Familial Aggregation of Hypertension Treatment and Control in the Genetic Epidemiology Network of Arteriopathy (GENOA) Study. *Am J Med*. 2004; **116(10)**: 676-681.

23. Kaplan E, Fein D, Morris R, Delis DC. WAIS-R NI Manual. San Antonio, Texas: Psychological Corporation, 1991.

24. Aryee MJ, Jaffe AE, Corrada-Bravo H, Ladd-Acosta C, Feinberg AP, Hansen KD et al. “Minfi: A flexible and comprehensive Bioconductor package for the analysis of Infinium DNA Methylation microarrays.” *Bioinformatics*, 2014; **30(10)**: 1363–1369.

25. Maksimovic J, Gordon L, Oshlack A. “SWAN: Subset quantile Within-Array Normalization for Illumina Infinium HumanMethylation450 BeadChips.” Genome Biology 2012; **13(6)**: R44

26. Ferrucci L, Bandinelli S, Benvenuti E, Di Iorio A, Macchi C, Harris TB, et al. Subsystems contributing to the decline in ability to walk: bridging the gap between epidemiology and geriatric practice in the InCHIANTI study. J Am Geriatr Soc, 2000; **48(12)**: 1618-25

27. Moore AZ, Hernandez DG, Tanaka T, Pilling LC, Nalls MA, Bandinelli S, et al. Change in Epigenome-Wide DNA Methylation Over 9 Years and Subsequent Mortality: Results From the InCHIANTI Study. J. Gerontol. A Biol. Sci. Med. Sci., 2016; **71(8)**: 1029-35.

28. Holly AC, Pilling LC, Hernandez D, Lee BP, Singleton A, Ferrucci L, et al. Splicing factor 3B1 hypomethylation is associated with altered SF3B1 transcript expression in older humans. Mech. Ageing Dev., 2014; **135**: 50-6.

29. Pidsley RY, Wong CC, Volta M, Lunnon K, Mil, J, Schalkwyk LC. A data-driven approach to preprocessing Illumina 450K methylation array data. BMC Genomics, 2013; **14**: 293.

30. Deary IJ, Whiteman MC, Starr JM, Whalley LJ, Fox HC. The impact of childhood intelligence on later life: following up the Scottish mental surveys of 1932 and 1947. *J Pers Soc Psychol*. 2004; **86(1)**: 130-147.

31. Deary IJ, Gow AJ, Taylor MD, Corley J, Brett C, Wilson V, et al. The Lothian Birth Cohort 1936: a study to examine influences on cognitive ageing from age 11 to age 70 and beyond. *BMC Geriatr*. 2007; **7**: 28.

32. Deary IJ, Gow AJ, Pattie A, Starr JM. Cohort profile: the Lothian Birth Cohorts of 1921 and 1936. *Int J Epidemiol*. 2012; **41(6)**: 1576-1584.

33. Wechsler D. Wechsler Memory Scale - Revised. New York, 1987

34. Lezak M. Neuropsychological testing. Oxford, UK, Oxford University Press, 2004

35. Nelson HE, Willison JR. National Adult Reading Test (NART) Test Manual (Part II). Windsor, UK: NFER-Nelson, 1991.

36. Wechsler D. WAIS-IIIUK administration and scoring manual. London, UK, Psychological Corporation, 1998

37. Shah S, McRae AF, Marioni RE, Harris SE, Gibson J, Henders AK, et al. Genetic and environmental exposures constrain epigenetic drift over the human life course. *Genome Res.*, 2014; **24(11)**: 1725-33.

38. Leveille SG, Kiel DP, Jones RN, Roman A, Hannan MT, Sorond FA et al. The MOBILIZE Boston Study: design and methods of a prospective cohort study of novel risk factors for falls in an older population. *BMC Geriatr* 2008; **8**: 16.

39. Rey GJ, Feldman E, Hernandex D, Levin BE, Rivas-Vazquez R, Nedd KJ, et al. Application of the multilingual aphasia examination-spanish in the evaluation of Hispanic patients post closed-head trauma. *Clin Neuropsychol* 2001; **15**: 13–18

40. Pugh KG, Kiely DK, Milberg WP, Lipsitz L. A. Selective impairment of frontal-executive cognitive function in african americans with cardiovascular risk factors. *J Am Geriatr Soc* 2003; **51**: 1439–1444

41. Royall DR, Mulroy AR, Chiodo LK, Polk MJ. Clock drawing is sensitive to executive control: a comparison of six methods*. J Gerontol B Psychol Sci Soc Sci* 1999; **54**: P328–33

42. Bell B, Rose CL, Damon A The Veterans Administration longitudinal study of healthy aging. *The Gerontologist* 1966; **6**: 179-184

43. Weisskopf MG, Wright RO, Schwartz J, Spiro A 3^rd^, Sparrow D, Aro A, et al. Cumulative lead exposure and prospective change in cognition among elderly men: the VA Normative Aging Study. *American Journal of Epidemiology* 2004; **160**: 1184-1193

44. Tombaugh TN, McIntyre NJ. The mini-mental state examination: a comprehensive review. *Journal of the American Geriatrics Society*. 1992; **40**: 922-935.

45. Triche TJ, Weisenberger DJ, Van Den Berg D, Laird PW, Siegmund KD, Low-level processing of Illumina Infinium DNA methylation beadarrays. *Nucleic acids research* 2013; **41**: e90-e90.

46. Teschendorff AE, Jones A, Fiegl H, Sargent A, Zhuang JJ, Kitchener HC, et al. Epigenetic variability in cells of normal cytology is associated with the risk of future morphological transformation. *Genome Med* 2012; **4**: 24.

47. Hofman A, Brusselle GG, Murad SD, van Duijn CM, Franco OH, Goedegebure A, et al. The Rotterdam Study: 2016 objectives and design update. *European journal of epidemiology*, 2015; **30(8)**: 661-708.

48. Hoogendam YY, Hofman A, van der Geest JN, van der Lugt A, Ikram MA. Patterns of cognitive function in aging: the Rotterdam Study. *European journal of epidemiology*, 2014; **29(2)**: 133-140.

49. Ibrahim-Verbaas CA, Bressler J, Debette S, Schuur M, Smith AV, Bis JC, et al. GWAS for executive function and processing speed suggests involvement of the CADM2 gene. *Molecular Psychiatry*, 2016; **21(2)**: 189-197.

50. Ligthart S, Steenaard RV, Peters MJ, van Meurs JB, Sijbrands EJ, Uitterlinden AG, et al. Tobacco smoking is associated with DNA methylation of diabetes susceptibility genes. *Diabetologia*, 2016; **59(5)**: 998-1006.

51. Moayyeri A, Hammond CJ, Valdes AM, Spector TD. Cohort Profile:

TwinsUK and healthy ageing twin study. *Int J Epidemiol,* 2013; **42**: 76-85.

52. Tsai PC, Van Dongen J, Tan Q., Willemsen G, Christiansen L, Boomsma DI, et al. DNA methylation changes in the IGF1R gene in birth weight discordant adult monozygotic twins. *Twin Res Hum Genet*, 2015; **18(6)**: 635-46.
