# Supplementary figures and images for: Meta-analysis of epigenome-wide association studies of cognitive abilities
Source: Mol Psychiatry. 2018 Jan 8;23(11):2133–44. doi: 10.1038/s41380-017-0008-y (PMC6035894; doi:10.1038/s41380-017-0008-y)

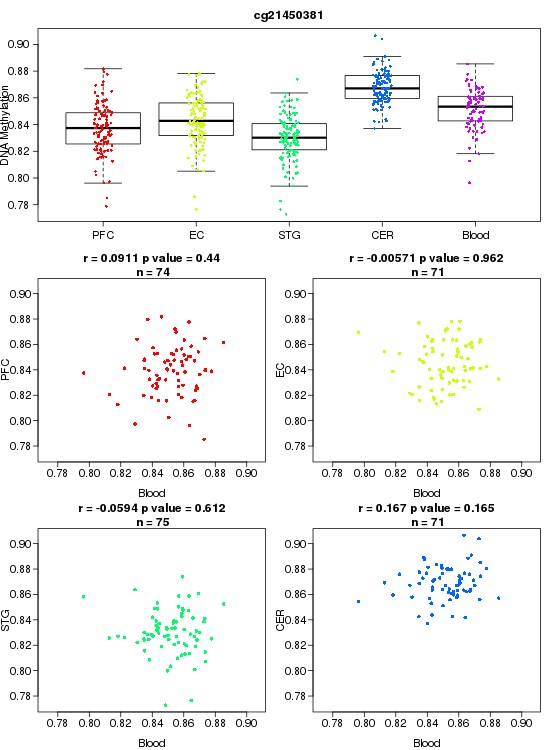

Supplement: Supplementary file 3 — Supplementary Figure 1 [file 41380_2017_8_MOESM3_ESM.jpg]

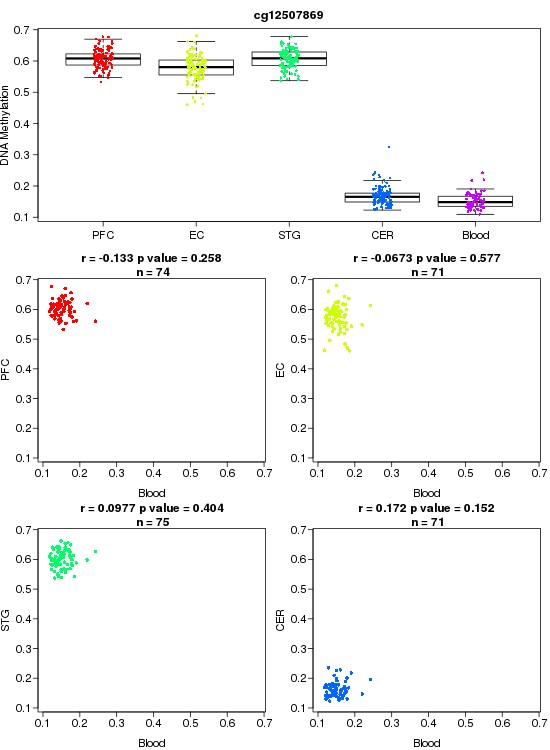

Supplement: Supplementary file 4 — Supplementary Figure 2 [file 41380_2017_8_MOESM4_ESM.jpg]
